# Determination of Flavonoid Glycosides by UPLC-MS to Authenticate Commercial Lemonade

**DOI:** 10.3390/molecules24163016

**Published:** 2019-08-20

**Authors:** Ying Xue, Lin-Sen Qing, Li Yong, Xian-Shun Xu, Bin Hu, Ming-Qing Tang, Jing Xie

**Affiliations:** 1 School of Pharmacy, Chengdu Medical College, Chengdu 610500, China; 2 Chengdu Institute of Biology, Chinese Academy of Sciences, Chengdu 610041, China; 3 Sichuan Provincial Center for Disease Control and Prevention, Chengdu 610041, China

**Keywords:** vortex-assisted dispersive liquid-liquid microextraction, flavonoid glycoside, UPLC-MS, counterfeiting lemonade

## Abstract

So far, there is no report on the quality evaluation of lemonade available in the market. In this study, a sample preparation method was developed for the determination of flavonoid glycosides by ultra-performance liquid chromatography–mass spectrometry (UPLC-MS) based on vortex-assisted dispersive liquid-liquid microextraction. First, potential flavonoids in lemonade were scanned and identified by ultra-performance liquid chromatography–time of flight mass spectrometry (UPLC-TOF/MS). Five flavonoid glycosides were identified as eriocitrin, narirutin, hesperidin, rutin, and diosmin according to the molecular formula provided by TOF/MS and subsequent confirmation of the authentic standard. Then, an ultra-performance liquid chromatography–triple quadrupole mass spectrometry (UPLC-QqQ/MS) method was developed to determine these five flavonoid glycosides in lemonade. The results showed that the content of rutin in some lemonade was unreasonably high. We suspected that many illegal manufacturers achieved the goal of low-cost counterfeiting lemonade by adding rutin. This suggested that it was necessary for relevant departments of the state to make stricter regulations on the quality standards of lemonade beverages.

## 1. Introduction

Lemon (*Citrus limon* L.) is considered the third most important citrus species in the world [1], with a large spectrum of biological activities that include antioxidant, antimicrobial, antiviral, antifungal, and antidiabetic activities [2,3], generating a large variety of healthy foods. Flavonoids are widely contained in lemon, conferring the typical taste and biological activities to lemon. According to the aglycone structures, flavonoids are divided into four classes: flavanones, flavones, flavonols, and flavans. Flavanones are the most abundant flavonoids, which are usually present in the 7-*O*-diglycoside form. Lemon flavanones are present in glycoside or aglycone forms. Among the phytochemicals, hesperetin and eriodictyol are the most abundant types of aglycones and rutinoside is the most abundant types of glycoside forms [4,5]. It has been reported that hesperidin and eriocitrin were the most abundant flavonoids in all the lemon juices studied and far exceed others [6,7,8].

Due to the high cost of fruit, counterfeiting of fruit juice has become a common problem in the industry. The three most common forms of counterfeiting are: (1) When a kind of cheaper fruit is used to replace all or part of it, (2) when a monomeric compound contained in the fruit with another cheaper source is added, and (3) when it is completely made up of additives such as artificial sweeteners, preservatives, and colors [9]. As the products produced with the first two counterfeiting methods contain some natural characteristic ingredients, they can generally meet the national testing standards [10]. However, such kinds of counterfeit juice not only seriously affect consumer confidence in the juice market, but may also cause a series of food safety problems. In addition to pure lemon juice, lemonade containing lemon ingredients occupies an increasing market share in the beverage market. Thus, it is of great scientific significance and commercial value to identify the authenticity of lemonade available in the market.

Some methods for analyzing lemon juice have been reported, such as nuclear magnetic resonance [11], ^13^C/^12^C isotope ratios [12], capillary electrochromatography (CEC) [13], and HPLC [6,7,14]. Among them, HPLC was considered as the most reliable method for determining flavonoids with high selectivity and sensitivity. Lemonade beverages currently available in the market contain a large number of additives besides a small amount of lemon juice. Therefore, a new sample preparation method is required to selectively separate and enrich low-content flavonoids from lemonade, so as to identify the authenticity of lemonade.

At present, sample preparation methods of flavonoids can be divided into liquid-liquid extraction (LLE) and solid phase extraction (SPE) [15,16,17]. However, they have some inherent disadvantages. For example, LLE needs a substantial amount of toxic solvents and is time-consuming. SPE materials are expensive and have poor reusability [18]. The dispersive liquid-liquid microextraction (DLLME) method developed in recent years can make up for these disadvantages [19,20,21]. DLLME can not only separate and enrich target analyte from aqueous solution, but also reduce or even eliminate the matrix interference of samples. Therefore, DLLME is considered to be an effective pretreatment method for food samples with the advantages of less solvent consumption, simple operation, high enrichment factor, etc. In order to improve the work efficiency by speeding up the mass transfer process and reducing the balance time, some assistant emulsification methods were also applied to improve the performance of DLLME, such as ultrasound-assisted [22], vortex-assisted [23], air-assisted [24], and microwave-assisted [25] DLLME. Currently, there are some studies on sample preparation of flavonoids by DLLME. However, as far as we know, there is no research on flavonoids in lemonade.

In this work, the sample preparation of flavonoids in lemonade was firstly performed by the vortex-assisted dispersive liquid-liquid microextraction (VA-DLLME) method. Then, the structure and content of flavonoids in lemonade available on the market from eight different manufacturers were identified and determined by ultra-performance liquid chromatography–time of flight mass spectrometry (UPLC-TOF/MS) and ultra-performance liquid chromatography–triple quadrupole mass spectrometry (UPLC-QqQ/MS), respectively. Finally, the counterfeiting phenomenon of lemonade was evaluated according to the determination results of flavonoids. As far as we know, this study was the first determination of flavonoid glycosides by UPLC-MS to authenticate commercial lemonade available in the market.

## 2. Results and Discussion

### 2.1. Identification of Flavonoid Glycosides by UPLC-TOF/MS

The time of flight mass spectrometer (TOF MS) was used to scan and identify potential flavonoids in lemonade for the first time in this work. As one of the most common high-resolution MS, TOF MS can determine the exact molecular formula of the target compound, thus identifying the structure in a complex matrix. After the target compound was located and identified, the triple quadrupole mass spectrometer (QqQ MS) was an excellent choice for subsequent quantitative analysis [26].

**Figure 1:**
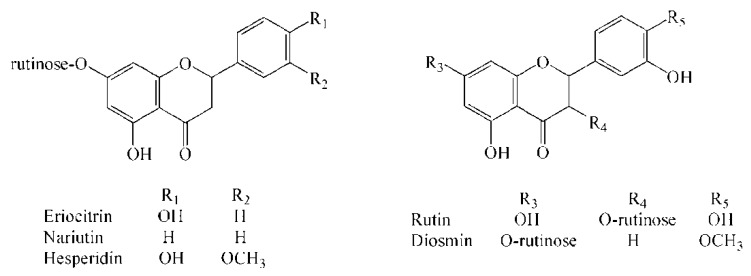
Chemical structures of eriocitrin, narirutin, hesperidin, rutin, and diosmin.

In this study, according to the calculation based on the molecular formula by TOF and the subsequent confirmation of the authentic standard under the same chromatographic conditions, 5 flavonoid glycosides in lemonade available in the market were located and identified (Figure 1), which were eriocitrin, narirutin, hesperidin, rutin, and diosmin, respectively. As shown in Table 1, the error of each compound in high-resolution MS is within ±5 ppm, which is the acceptable error limit for structure confirmation [27].

### 2.2. The Selection of VA-DLLME Conditions

**Figure 2:**
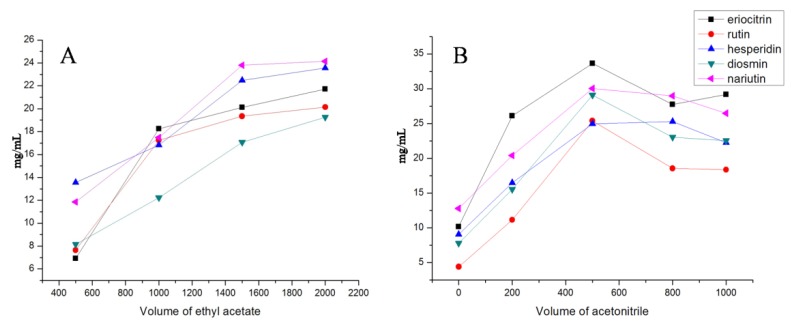
The evaluation of extraction conditions of the amount of ethyl acetate (**A**) and acetonitrile (**B**).

Since the extraction conditions have a crucial influence on the performance of VA-DLLME, single-factor experiments were carried out to select the extraction conditions of the amount of ethyl acetate and acetonitrile. In the present study, recoveries of 5 flavonoid glycosides were assessed by means of fixing one variable and changing the other two variables. The results are shown in Figure 2. Due to structural differences, the recoveries of the 5 flavonoid glycosides were different, but the overall trend was relatively consistent. Based on the investigation of single-factor experiments, the VA-DLLME condition was set as 1 mL of lemonade, 500 µL acetonitrile, and 1.5 mL ethyl acetate.

### 2.3. Determination of Flavonoid Glycosides by UPLC–QqQ/MS

**Figure 3:**
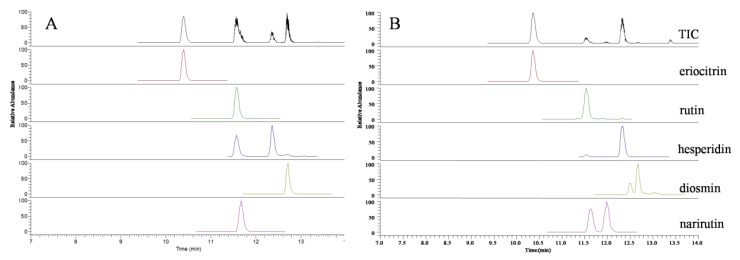
UPLC–QqQ/MS total ion count chromatograms of five flavonoid glycosides standards (**A**) and commercial lemonade sample (**B**).

All 5 flavonoid glycosides are acidic compounds. Therefore, acid mobile phase could increase the separating degree, symmetry factor, and the number of theoretical plates. Considering the ion suppression induced by a high concentration of acid, 0.2% formic acid was finally added into the mobile phase [28]. In order to optimize the MS condition of 5 flavonoid glycosides in the present study, all of these target analytes were tested in direct infusion mode using the full-scan MS method, respectively. It was found that the negative mode was more sensitive and selective than the positive mode. By optimizing mass spectrum variables, including the vaporizer temperature, sheath gas pressure, aux gas pressure, the parent/product ion pairs, collision energy, and S-Lens value, two stable product ions with high sensitivity were selected for MRM analysis (Table 1). The representative mass spectra of lemonade samples are shown in Figure 3.

### 2.4. Speculation on the Possible Counterfeiting Means of Lemonade

A total of 8 batches of lemonade samples purchased from local supermarkets was determined by the proposed UPLC-QqQ/MS method. The contents of 5 flavonoid glycosides are shown in Table 2. The content of total flavonoid glycosides in lemonade varies greatly. On the surface, it seems that the higher the content of total flavones, the higher the amount of lemon juice added in lemonade, which means the better the quality of the product. However, after further analysis of the content of monomeric compounds, it was found that the main ingredients in S1–S4 were flavanone glycosides (mainly hesperidin and eriocitrin) and the content of flavonol glycosides (mainly rutin) was relatively low. This result is consistent with the distribution characteristics of flavonoid glycosides in *Citrus* L. With regard to S5–S8, the content of rutin is extremely high and hesperidin as a characteristic ingredient of *Citrus* L. is not detected (nd). Hesperidin was the predominant flavonoid glycoside in lemon reported by the previous study. For example, Mannan et al. reported values of 67 ± 15 mg/L for hesperidin in 38 natural lemon juices, showing that the absence of this compound in lemonade shows it to be a possible counterfeit [29]. Under normal circumstances, the content of rutin in lemon should not exceed the content of hesperidin. Xi reported the contents of hesperidin and rutin in juice varied from 105.5 to 210.3 µg/g and nd to 3.82 µg/g, respectively [30]. Due to the abnormal phenomenon in our work, we have reason to suspect that S5–S8 were counterfeited as there was no or only a small trace of lemon juice and had instead a large amount of rutin added to meet the national testing standards (colorimetric assay by UV-Vis) of fruit juice products. Rutin is widely distributed in the plant kingdom. It was reported that its content in *Sophora japonica* L. was up to 37.8% [31]. Therefore, only with a simple separation process the commercialized low-cost supply of rutin can be realized [32]. For example, the price of rutin reagent supplied by Aladdin is ￥368/100 g and if it is a crude extract of food-grade, the price will be even lower. According to the testing method of total flavonoids in fruit juice beverage specified by national standard, rutin also has an obvious response in the colorimetric assay by UV-Vis at a wavelength of 420 nm. Therefore, illegal businessmen achieved the goal of low-cost counterfeiting lemonade by adding rutin.

## 3. Material and Methods

### 3.1. Chemicals and Reagents

A total of eight lemonade samples were purchased from local supermarkets. A total of five authentic standards of eriocitrin, narirutin, hesperidin, rutin, and diosmin were obtained from Chengdu Push Bio-technology Co., Ltd. (Chengdu, China). The Milli-Q water purification system was used to prepare ultra-pure water for UPLC analysis (Millipore, Bedford, MA, USA). Formic acid and acetonitrile of LC/MS grade for UPLC-MS analysis were purchased from Sigma-Aldrich. Ethyl acetate, ether, dichloromethane, methanol, acetone, and acetonitrile of analytical grade were purchased from Sinopharm Chemical Reagent Co., Ltd. (Shanghai, China).

### 3.2. Preparation of Standard Solution

Stock solutions of five target analytes (eriocitrin, narirutin, hesperidin, rutin, and diosmin) were prepared by dissolving each 10 mg authentic standard in 10 mL of methanol. Then, 250 μL of each of the five stock solutions was transferred to a 50-mL volumetric flask and diluted with 20% methanol to obtain the mixed stock solution. Next, 500 μL of mixed stock solution was transferred to a 50-mL volumetric flask and diluted with 20% methanol to obtain the working solution I with a concentration of approximately 50 ng/mL. Finally, mixed working solutions II–V were obtained by diluting working solution I with respective concentrations of about 20.0 ng/mL, 10.0 ng/mL, 5.0 ng/mL, and 2 ng/mL. All the solutions were stored in a refrigerator at 4 °C before use.

### 3.3. Sample Preparation by the VA-DLLME Procedure

Accurately add 1 mL of lemonade to a 4 mL centrifuge tube, then add 500 µL of acetonitrile and 1.5 mL ethyl acetate, then vortex for 30 s. After centrifugation, the upper organic phase was transferred. The extraction was repeated once using another 1.5 mL of ethyl acetate and the combined solvent of the upper organic phase was removed by a Termovap Sample Concentrator. The resulting residue was re-dissolved in 1 mL of 20% methanol and filtered through a 0.22 µm filter for UPLC-MS analysis.

### 3.4. UPLC–MS Analysis

#### 3.4.1. Identification of Flavonoid Glycosides by UPLC-TOF/MS

The Shimadzu UPLC ((Shimadzu, Kyoto, Japan) system consists of an online degasser (DGU-20A5R), an auto-sampler (SIL-30AC), two pumps (LC-30AD), and a column oven (CTO-30aHE). Chromatographic separation was performed on a Waters BEHC18 analytical column (2.1 × 100 mm, 1.7 μm, Waters, Milford, MA, USA) at 40 °C. The mobile phase consisted of 0.2% formic acid and acetonitrile. The linear gradient elution with a constant flow rate of 0.2 mL/min was 10%~10%~40%~95%~10% acetonitrile at 0~1~10~13~15 min. The sample solution and mixed working solutions of 5 µL were injected into the UPLC system by the auto-sampler.

TOF/MS measurements in negative ion mode were performed on a 4600 Q-TOF mass spectrometer (AB Sciex, Concord, CA, USA) equipped with an electrospray ionization (ESI) source with the following parameters: Ion source gas 1 (GS1) at 50 psi, ion source gas 1 (GS1) (N_2_) at 50 psi, curtain gas at 35 psi, temperature at 500 °C, and ionspray voltage floating at −4500 V. The mass range was set to *m*/*z* 100–800. The system was operated under Analyst 1.6 and Peak 2.0 (AB Sciex, Concord, CA, USA) and used an APCI negative calibration solution to calibrate the instrument’s mass accuracy in real-time.

#### 3.4.2. Determination of Flavonoid Glycosides by UPLC-QqQ/MS

Chromatographic separation was the same as that used in UPLC-TOF/MS analysis described above. QqQ/MS measurements in negative ion mode were accomplished by a triple quadrupole mass spectrometer equipped with an ESI source (Thermo Fisher Scientific, San Jose, CA, USA). The determination of the target analytes was performed in a multi-reaction monitoring mode. The MS parameters were as follows: Vaporizer temperature and capillary temperature both 350 °C, aux gas pressure of 10 Arb, sheath gas pressure of 40 Arb, ion sweep gas pressure of 2 Arb, discharge current of 4.0 µA, and spray voltage of −2000 V. Data collection and processing were conducted with Thermo Xcalibur Workstation (Version 2.2, Thermo).

### 3.5. Analytical Figures of Merit

Method validation was performed according to the above UPLC–QqQ/MS conditions. After it was determined by the mixed working solutions I–V, the calibration curves of five analytes were obtained as shown in Table 3 by taking the concentration of each authentic standard as the abscissa (x) and the corresponding peak area as the ordinate (y), respectively. The limit of detection (LOD) and the limit of quantification (LOQ) were measured by a gradual dilution process of the standard stock solutions until the signal-to-noise ratio of 3:1 and 10:1, respectively. The precision was evaluated by standard working solution III, which was tested within one day to determine the intra-day precision and was tested within 3 days to determine the inter-day precision. The repeatability was evaluated by analyzing six independent portions of sample S4 with parallel running. The recovery was carried out by spiking an amount of about 1:1 of authentic standards to six independent portions of sample S4 with parallel running. The validation results are summarized in Table 3, which show that the present developed UPLC–QqQ/MS method meets the requirements of quantitative analysis and was appropriate for the determination of five flavonoid glycosides in lemonade. The analytical figures of merit were compared with those of several other quantitative methods reported for flavonoid glycosides in lemon as shown in Table 4.

## 4. Conclusions

In this study, five flavonoid glycosides of eriocitrin, narirutin, hesperidin, rutin, and diosmin in lemonade were identified and determined by UPLC-TOF/MS and UPLC-QqQ/MS, respectively. By estimating the content characteristics of flavonoid glycosides in the samples, we highly suspected that some lemonade available in the market was counterfeited: Cheap rutin was added to increase the content of “total flavonoids of lemon”. This indicates that besides using total flavonoids, the content of multiple flavonoid compounds should be included in the quality standard of lemonade in the future.

## Figures and Tables

**Table 1:** UPLC-MS parameters of five analytes in the negative ion-scan mode.

Analyte	TOF/MS			QqQ/MS	
	Quasi-Molecular Ion (*m*/*z*)	Error (ppm)	Product Ion (*m*/*z*)	Parent Ion (*m*/*z*)	Product Ion (*m*/*z*)
eriocitrin	595.16788	1.7	287.0586, 151.0065	595	287 *, 151 ^#^
narirutin	579.17238	0.8	271.0612	579	271 *, 151 ^#^
hesperidin	609.18386	2.2	301.0737	609	301 *, 286 ^#^
rutin	609.14689	1.3	301.0383, 300.0281	609	300 *, 271 ^#^
diosmin	607.16784	1.0	299.0582, 284.0345	607	299 *, 284 ^#^

Note: * quantitative ion, ^#^ qualitative ion.

**Table 2:** The contents of five flavonoid glycosides in eight lemonade samples (µg/100 mL).

Sample No.	Eriocitrin	Rutin	Hesperidin	Diosmin	Narirutin	Total
S1	0.04	0.27	1.00	nd	nd	1.31
S2	0.92	0.34	3.82	0.66	nd	5.75
S3	1.73	0.29	16.33	2.95	nd	21.30
S4	28.96	6.01	28.30	0.28	0.74	49.07
S5	2.66	191.54	nd	0.64	nd	194.83
S6	0.04	243.71	nd	nd	nd	243.75
S7	0.05	264.24	nd	nd	nd	264.29
S8	0.35	470.00	nd	nd	nd	470.35

**Table 3:** The results of method validation.

Analyte	Regression Equation	Linear Range (ng/mL)	LOD	LOQ	Precision (RSD, *n* = 6)		Repeatability (*n* = 6)		Recovery (*n* = 6)	
	(y = ax + b, r^2^)		(ng/mL)	(ng/mL)	Intra-Day	Inter-Day	Mean (µg/100 mL)	RSD	Mean	RSD
eriocitrin	y = 523.81x − 82.61, 0.996	2.01–50.3	0.70	2.01	1.36%	3.53%	28.9	3.22%	88.5%	3.93%
rutin	y = 1034.77x − 1160.22, 0.996	2.44–60.9	0.81	2.44	2.51%	3.79%	6.01	4.62%	89.9%	4.51%
hesperidin	y = 513.03x + 252.28, 0.998	2.01–50.4	0.70	2.10	1.97%	2.58%	28.3	3.47%	88.7%	5.30%
diosmin	y = 769.76x + 84.74, 0.995	2.14–53.4	0.71	2.14	2.02%	3.17%	0.28	5.47%	102%	2.61%
narirutin	y = 556.25x + 148.69, 0.997	2.02–51.2	0.70	2.02	1.48%	3.69%	0.74	4.86%	92.8%	4.47%

**Table 4:** Comparison of analytical methods reported for determination of flavonoid glycosides in lemon.

Method	Analyte	Linea Range	LOD	LOQ	Recovery
CEC [13]	eriocitrin, narirutin, hesperidin	5–200 μg/mL	2.5 μg/mL	5 μg/mL	71–112%
HPLC/UV [33]	narirutin, hesperidin, diosmin	0.25–20 μg/mL	-	0.1 μg/mL	-
HPLC/UV [34]	narirutin	2–50 mg/L	1.25 mg/L	2.5 mg/L	83%
	hesperidin	2–50 mg/L	1.0 mg/L	2.5 mg/L	74%
HPLC/UV [35]	eriocitrin	1.01–50.50 μg/mL	0.02 μg/mL	0.065 μg/mL	103.10%
	narirutin	0.505–10.10 μg/mL	0.024 μg/mL	0.18 μg/mL	99.14%
	hesperidin	5.00–100.00 μg/mL	0.04 μg/mL	0.132 μg/mL	99%
	rutin	0.101–10.100 μg/mL	0.079 μg/mL	0.263 μg/mL	98.37%
UPLC/UV [36]	eriocitrin	0.5–130 mg/L	6 μg/kg	-	90.50%
	narirutin	0.05–300 mg/L	5 μg/kg	-	87.40%
	hesperidin	0.05–500 mg/L	8 μg/kg	-	92.70%
	rutin	0.05–310 mg/L	5 μg/kg	-	88.40%
	diosmin	0.01–200 mg/L	8 μg/kg	-	100.80%
